# Spatial clustering and transmission networks of multidrug-resistant tuberculosis in Rwanda: a national retrospective genomic and spatial epidemiological study

**DOI:** 10.1136/bmjopen-2025-111823

**Published:** 2026-08-26

**Authors:** Isabel Cuella-Martin, Francois Hakizayezu, Wim Mulders, Hosee Niyompano, Docteur Runyambo, Jelle Keysers, Willem B De Rijk, Yves Habimana Mucyo, Patrick Migambi, Claude Mambo Muvunyi, Conor J Meehan, Bouke C de Jong, Leen Rigouts, Jean Claude Semuto Ngabonziza, Ellen Mitchell

**Affiliations:** 1 Unit of Mycobacteriology, Department of Biomedical Sciences, Institute of Tropical Medicine, Antwerp, Belgium; 2 Department of Biomedical Sciences, University of Antwerp, Antwerp, Belgium; 3 National Reference Laboratory Division, Rwanda Biomedical Center, Kigali, Rwanda; 4 Tuberculosis and Other Respiratory Diseases Division, Rwanda Biomedical Center, Kigali, Rwanda; 5 Rwanda Biomedical Center, Kigali, Rwanda; 6 Nottingham Trent University, Nottingham, UK; 7 Research Innovation and Data Science Division, Rwanda Biomedical Center, Kigali, Rwanda; 8 Department of Clinical Biology, University of Rwanda, Kigali, Rwanda; 9 Unit of Mycobacterial Diseases and Neglected Tropical Disease, Department of Public Health, Institute of Tropical Medicine, Antwerp, Belgium

**Keywords:** Tuberculosis, Epidemiology, Public health

## Abstract

**Abstract:**

**Background:**

Approximately 96% of rifampicin resistance/multidrug-resistant tuberculosis (RR/MDR-TB) cases in Rwanda result from direct transmission rather than acquired resistance. However, the nationwide spatial distribution and transmission dynamics of RR/MDR-TB remain poorly characterised. This study aims to analyse spatial patterns of RR/MDR-TB in Rwanda and explore relationships between spatial proximity and RR/MDR-TB strains’ genetic relatedness.

**Methods:**

We conducted a retrospective analysis of 249 confirmed RR-TB cases across Rwanda from 2017 to 2024, using the known geolocations of patients’ residences. Spatial and space-time clustering was assessed using Kulldorff’s scan statistics. Demographic and socioeconomic determinants were evaluated using multivariable regression. For 201 cases with whole-genome sequencing data, we performed transmission analysis using a 5-SNP threshold to define recent transmission clusters and investigated spatial relationships within genetically related strains.

**Results:**

Significant spatial clustering of RR/MDR-TB was identified in 21 sectors, mainly in Nyarugenge, southern Gasabo and western Kicukiro (relative risk: 10.06; p<0.001). Our multivariable analysis showed that population density is positively associated with case notification rates. Molecular analysis revealed 88.5% of cases belonged to genotype clusters defined using a 12-SNP threshold, with 73.6% forming clusters at a strict 5-SNP threshold. Spatial K-function analysis of the six major clusters revealed heterogeneous transmission patterns, characterised by both tightly clustered outbreaks and regional transmission networks that spanned administrative boundaries. Most clusters (5/6) extended beyond Kigali, indicating that transmission networks operate across administrative divides.

**Conclusion:**

RR/MDR-TB in Rwanda shows significant spatial clustering with transmission occurring through both localised and regional networks. Integrating genomic and spatial data reveals transmission patterns that extend beyond household contacts and administrative boundaries. These findings underscore the need to implement geographically targeted interventions that address community-level transmission to control RR/MDR-TB in Rwanda effectively.

**STRENGTHS AND LIMITATIONS OF THIS STUDY**

- To characterise rifampicin resistance/multidrug-resistant tuberculosis (RR/MDR-TB) transmission dynamics, whole genome sequencing was integrated with village-level residential data at a national level.
- Complementary binomial regression models were used to assess potential drivers of RR/MDR-TB distribution and validate that identified hotspots reflected genuine epidemiological clustering rather than detection bias.
- Whole-genome sequencing with a 5-SNP threshold combined with K-function spatial analysis allowed high-resolution characterisation of transmission clusters and their geographic structure.
- The absence of individual-level epidemiological data limited the ability to confirm direct person-to-person transmission within identified genotype clusters.
- Incomplete sampling coverage, primarily due to culture failures and insufficient DNA yield, may have resulted in undetected transmission clusters and underestimation of the true extent of transmission networks.

## Introduction

 Multidrug-resistant tuberculosis (MDR-TB), defined as TB that is resistant to at least rifampicin and isoniazid, continues to be a significant challenge in the management of TB. In 2023, an estimated 400 000 individuals worldwide developed rifampicin resistance (RR)/MDR-TB, with an associated 150 000 deaths.^1^ Research shows that in most MDR-TB high-burden settings, direct transmission accounts for up to 95% of cases, rather than the selection of de novo resistance during previous TB treatment.^2–4^ Identifying where, how and who is exposed to circulating MDR-TB bacilli is crucial for developing effective control strategies.^5^

In Rwanda, 1.2% of new patients with TB and 3.4% of previously treated patients were diagnosed with MDR-TB in 2023.^1^ Although Rwanda has achieved high coverage of drug-susceptibility testing, with 96% of new TB cases and 94% of previously treated cases undergoing RR testing, a substantial gap remains between the estimated number of incident MDR-TB cases and those detected.^1 6^ This discrepancy may be partially explained by false-RR GeneXpert Ultra results, which led to an overestimation of the MDR-TB burden.^7^ Notably, previous studies in Rwanda determined that around 96% of MDR-TB cases are due to direct transmission, with a large proportion belonging to a single dominant clone (Rwanda rifampicin-resistant clone (R3clone)).^4^ The predominance of transmission-driven MDR-TB highlights the need for targeted active case-finding efforts that disrupt transmission hotspots. However, precision targeting is particularly challenging in low-burden settings where the number needed to test to find one actual case is often high.^8^

The spatial distribution of MDR-TB is rarely homogeneous across geographic regions. Studies have shown that MDR-TB clusters geographically and is linked to factors such as specific neighbourhood characteristics, socioeconomic status and population density.^9 10^ Multiple environmental risk factors influence these spatial patterns, including urban overcrowding, limited healthcare access, HIV co-infection, poverty-related housing conditions, environmental factors like ventilation quality and social determinants such as incarceration rates and migration patterns.^11–13^ Identification of MDR-TB hotspots is important to improve the precision of the interventions and optimise the utilisation of scarce resources. However, areas of high MDR-TB burden may reflect a higher risk of progression to active disease rather than a higher risk of transmission.^14^ Reductions in costs associated with genomic sequencing and the collection of detailed spatial data have accelerated the application of phylogeographic analyses for understanding the transmission of *Mycobacterium tuberculosis* complex (MTBC).^15–18^ The integration of spatial and genomic analysis can reveal patterns of active transmission that otherwise go unobserved.

Despite research on the contribution of primary versus acquired resistance to the prevalence of RR/MDR-TB in Rwanda, no studies have yet explored the geographic distribution of RR/MDR-TB transmission at the national level.^4^ This study aims to: (1) analyse the spatial and temporal patterns of RR/MDR-TB in Rwanda over 8 years; (2) evaluate the influence of socioeconomic and demographic factors on the spatial distribution of RR/MDR-TB and (3) explore relationships between spatial proximity and strains’ genetic relatedness to reconstruct likely active transmission chains and determine whether they occur within distinct geographic areas.

## Methods

### Study setting and population

Rwanda, a country in East Africa with a surface area of 26 338 km², is administratively divided into five provinces, 30 districts, 416 sectors, 2148 cells, and, ultimately, into 14 837 villages. As of 2022, the estimated population was 13,246,394, with an average population density of 503 inhabitants per km^2^, ranking third on the African continent. The highest population density was recorded in Kigali City, with 2403 inhabitants per km².^19^ The country’s overall sex ratio is 94.3 males per 100 females (48.5% men), with Kigali City having a higher male proportion than the rest of the country (50.9%) due to rural-urban migration patterns among young men.^20^ This demographic distribution may affect MDR-TB transmission, as reflected in the male-to-female ratio of 2.4 among RR-TB cases in the 2023–2024 fiscal year.^21^ HIV remains a significant comorbidity, with 18.5% of MDR-TB cases being HIV-positive.^21^ This coinfection impacts treatment outcomes, with success rates of 80% in patients with HIV-positive MDR-TB compared with 91% in patients with HIV-negative.^21^

**Figure 1:**
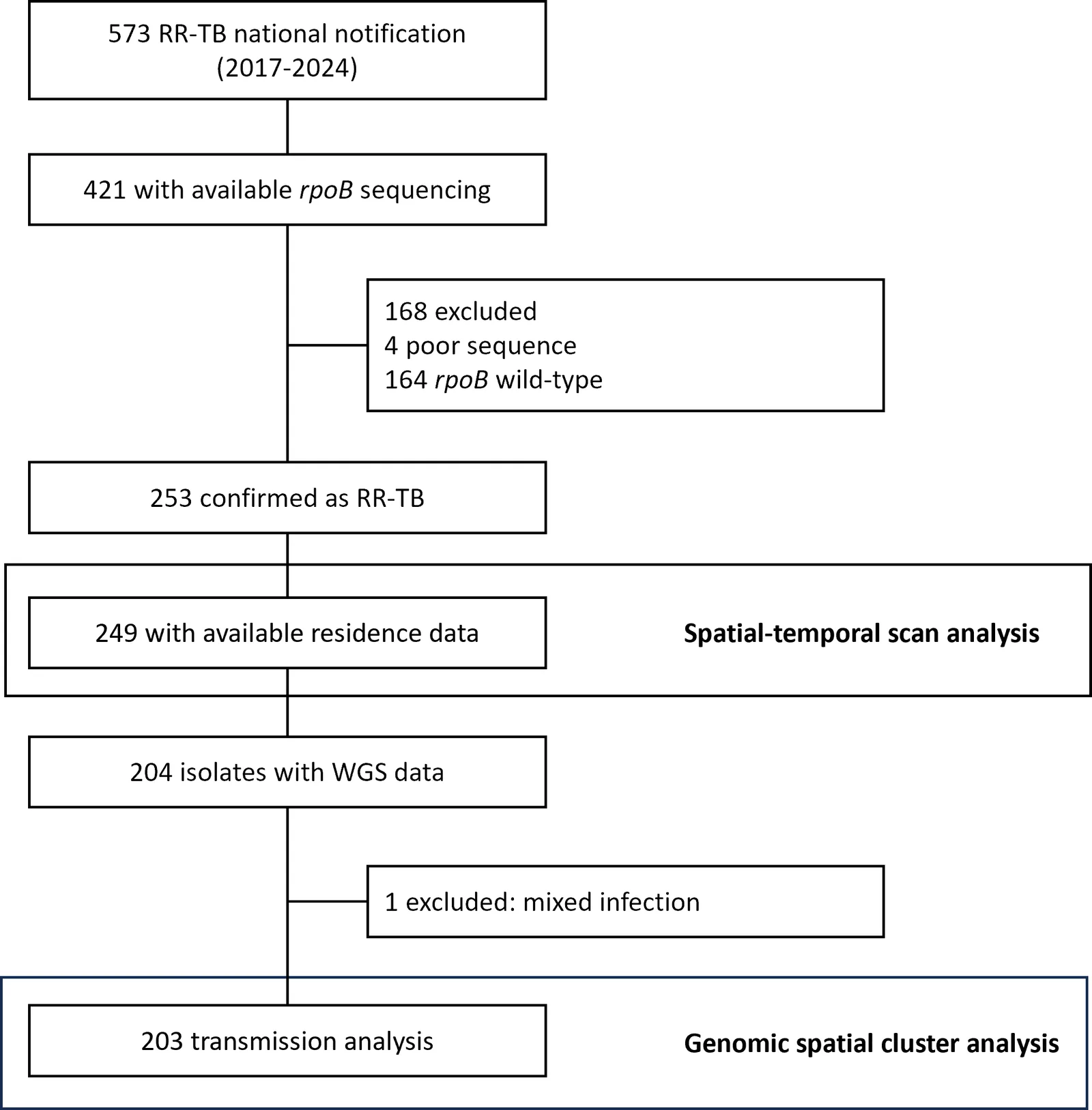
Flowchart showing the inclusion and exclusion of samples for both spatial-temporal scan analysis and molecular epidemiology analysis. The reduction from 249 cases with residence data to 204 cases with WGS was primarily due to culture failures. For the remaining 45 cases, rpoB mutations were detected through Sanger sequencing or Deeplex testing rather than WGS. RR-TB, rifampicin-resistant tuberculosis; WGS, whole genome sequencing.

This study analysed routinely collected data on initially diagnosed RR/MDR-TB cases identified across Rwanda between 1 January 2017 and 31 December 2024 (online supplemental table S1; figure 1). Both actively and passively detected cases were included and linked to geographic and contextual characteristics to conduct a spatial and temporal ecological assessment. During this period, the GeneXpert MTB/RIF (Xpert) and GeneXpert MTB/RIF Ultra (Ultra) assays (Cepheid, Sunnyvale, CA) were used as first-line diagnostic tools for all presumptive patients with TB in Kigali province.^7 22^ Additionally, GeneXpert was used nationwide to detect RIF resistance in patients with microscopically diagnosed TB and for TB detection among patients categorised as high-risk and priority groups, such as people with HIV, children, contacts of patients with TB and prisoners.^7 22^ Patients diagnosed with RR/MDR-TB nationwide are admitted to the RR/MDR-TB clinic, where, before initiating RR/MDR-TB treatment, three additional sputum samples were collected for further testing. Two of these samples were processed separately for mycobacterial culture at the National Reference Laboratory.^22^ Positive cultures were confirmed by an immunochromatographic test (SD MPT64TB Ag kit; SD Bioline, Seoul, South Korea). When a positive culture was available, whole-genome sequencing (WGS) was performed on gDNA, as described previously.^7 22^ Sanger sequencing of the *rpoB* gene was performed on remnants of the second sputum sample.^23^ If tests on the sputum sample did not produce a result, the tests were repeated on the corresponding culture isolate when available. Additionally, some cases underwent next-generation sequencing by Deeplex-MycTB (Deeplex; GenoScreen, Lille, France) on sputum DNA extracts using either sample two or three, as part of another study.^22^ In this study, individuals were designated as RR/MDR-TB when a resistance-associated mutation was detected in the *rpoB* gene by any of the aforementioned sequencing methods.

### Data collection and variable definitions

Clinical information, including sex, age, history of TB, as well as individual-level residential addresses, was collected during the interview conducted by health nurses on admission to the RR/MDR clinic. RR/MDR-TB data were aggregated to the sector level to obtain case notification rates and conduct ecological analyses, as most demographic and socioeconomic variables were available at this administrative level. For molecular transmission analysis, village-level geographic data were used to provide higher spatial resolution for examining relationships between genetic relatedness and geographic proximity.

Nationwide demographic and socioeconomic variables, including population data, population density (per 1000 people per km²) and gender ratio (male to female ratio), were derived from the 5th Rwanda population and housing census (PHC) conducted in August 2022 and provided by the National Institute of Statistics of Rwanda, Ministry of Finance and Economic Planning of Rwanda. We used the Relative Wealth Index (RWI) from Data for Good (Meta, Menlo Park, CA) to represent economic status across Rwanda’s sectors. This public dataset provides high-resolution estimates of relative poverty and wealth by applying machine-learning algorithms to multiple data sources, including satellite imagery and mobile phone network data. The investigation rate was defined as the total volume of smear microscopy and GeneXpert tests performed within a district divided by the district’s population. This district-level rate was then applied uniformly to all sectors within that district to account for cross-sector healthcare utilisation patterns. The location and occupancy rate of correctional facilities were obtained from the annual 2022–2023 report of the National Commission for Human Rights of Rwanda.

### Spatial and spatial-temporal clustering analysis

The following section describes the ecological spatial analyses conducted to identify RR/MDR-TB hotspots and their sociodemographic determinants at the sector level.

For graphical analysis, the incidence of RR/MDR-TB was described per sector for every 100 000 inhabitants. Spatial autocorrelation was explored using the global Moran’s I statistic. To analyse the spatial distribution of RR/MDR-TB cases and identify significant space-time and purely spatial clusters, we employed a discrete Poisson model using Kulldorff’s spatial scan statistic software (SaTScan, V.10.1.2, Kulldorff, Boston, MA).^24^ The analysis used circular (purely spatial) or cylindrical (space-time) clusters with a maximum size of 50% of the population. Only statistically significant clusters (p<0.05) were interpreted, with p values determined through Monte Carlo testing. For the space-time analysis, the number of notifications per year was used, while for the purely spatial analysis, the total number of notifications over the entire 8-year study period was considered. In both cases, the estimated population per sector from the 2022 PHC was used as the reference population.

To examine potential drivers of RR/MDR-TB distribution and account for confounding factors, we conducted two complementary regression analyses. First, to identify factors associated with RR/MDR-TB case notification rates across all sectors, we performed a Poisson regression with the sector-level case notification rate as the dependent variable. Second, to determine whether specific factors predicted inclusion in hotspots, we conducted a binomial regression analysis. Factors that were statistically significant or identified as relevant in the literature were included in multivariable models. Before building these models, we assessed multicollinearity between predictor variables using variance inflation factors, which ranged from 1.3 to 4.7, indicating acceptable levels of correlation between predictors. To address potential detection bias from differential test access, we included the investigation rate as a covariate. Statistical significance was set at p<0.05. All sensitivity analyses were conducted using R statistical software (V.4.4.2, R Core Team, Vienna, Austria), and QGIS (V.3.28, QGIS.ORG, Laax, Switzerland) was used for graphical visualisation.^25 26^

### Molecular epidemiology

The following section describes the molecular epidemiological analyses used to characterise transmission clusters, followed by the spatial analyses applied to examine the geographic structure of these genetically defined clusters.

For the molecular epidemiology analysis, only patients who had both WGS and residential data were considered, excluding those with a possible mixed-strain infection, defined as polyclonal TB infection (ie, multiple strains in one sample) or heteroresistance (ie, mixed population of drug-resistant mutants and wildtype, when the mutant population is still <95%) (figure 1). To discard potential selection bias, demographic and clinical characteristics of WGS-available and WGS-not available cases were compared using Fisher’s exact test for categorical variables and the Wilcoxon rank-sum test for age (table 1). For the initial phylogenetic analysis and cluster determination, we included both new and previously treated TB cases to fully characterise the genetic diversity of RR/MDR-TB strains circulating in Rwanda. A SNP alignment was performed using MTBseq (V.1.1.0, Research Center Borstel, Borstel, Germany), applying a 5-SNP threshold to define transmission clusters.^27^ A maximum-likelihood phylogenetic tree was built with RAxML-NG (V.1.2.0, HITS gGmbH, Heidelberg, Germany).^28^ The run used a GTR+G+ASC substitution model with site-repeat optimisation, incorporating 10 starting trees and 200 bootstrap replicates for stable tree construction.^29^ Analysis focused on six clusters (≥10 individuals with a shared genotype), with ungrouped cases (those not belonging to any cluster ≥10 individuals) as a comparison group to represent the underlying density of RR/MDR-TB infection in the population. To increase resolution, geographic locations were mapped using village-level data; where unavailable, cell-level centroids were used. For recent transmission analysis, we restricted our dataset to new TB cases only, as these are more likely to represent recent transmission events rather than relapses or treatment failures. Spatial clustering was assessed using K-function analysis, adjusting for population density.^30^ We used 999 permutations to generate 95% CIs, identifying significant clustering when values fell outside these bounds. Spatial-temporal trends were explored by measuring distances between the first and subsequent diagnosed cases in each group. Analysis and data visualisation with ggplot2 was performed in R statistical software V.4.4.2.^25 31^ The spatstat and sp packages were used for K-function analysis, and sf and geosphere were used for calculating distance to the index case.^32–35^ We used R packages ape, phytools and mapdata to annotate and visualise the tree with geographical data.^36–38^ The main phylogenetic tree was annotated using the Interactive Tree Of Life (iTOL, V.6, EMBL, Heidelberg, Germany) software.^39^

**Table 1:** Demographic and clinical characteristics of patients with RR-TB included in the spatial-temporal scan analysis (n=249), stratified by availability of whole genome sequencing (WGS) data, 2017–2024

Category	All cases (n=249)	WGS-available (n=201)	WGS-not available (n=48)	P value
	n (%)	n (%)	n (%)	
Gender				0.668
Male	182 (73.1)	145 (72.1)	37 (77.1)	
Female	66 (26.5)	55 (27.4)	11 (22.9)	
Unknown	1 (0.4)	1 (0.5)	0 (0.0)	
Age				0.180
<25	26 (10.4)	22 (10.9)	4 (8.3)	
25–44	154 (61.9)	126 (62.7)	28 (58.3)	
45–64	60 (24.1)	44 (21.9)	16 (33.3)	
≥65	8 (3.2)	8 (4.0)	0 (0.0)	
Unknown	1 (0.4)	1 (0.5)	0 (0.0)	
Median (IQR)	37 (29–45)	37 (29–45)	40 (30–49)	
HIV status				0.380
Positive	73 (29.3)	62 (30.8)	11 (22.9)	
Negative	172 (69.1)	135 (67.2)	37 (77.1)	
Unknown	4 (1.6)	4 (2.0)	0 (0.0)	
TB history				0.284
New case	192 (77.1)	151 (75.1)	41 (85.4)	
Retreatment case	52 (20.9)	45 (22.4)	7 (14.6)	
Unknown	5 (2.0)	5 (2.5)	0 (0.0)	
Province				0.305
Kigali City	119 (47.8)	99 (49.3)	20 (41.7)	
East	38 (15.3)	32 (15.9)	6 (12.5)	
North	16 (6.4)	10 (5.0)	6 (12.5)	
South	49 (19.7)	40 (19.9)	9 (18.8)	
West	27 (10.8)	20 (10.0)	7 (14.6)	

P values compare WGS-available and WGS-not available cases.

RR-TB, rifampicin-resistant tuberculosis; TB, tuberculosis.

## Results

### Dataset overview

Between 2017 and 2024, 573 nationwide cases of RR/MDR-TB were initially diagnosed using Xpert MTB/RIF (figure 1). Of these, 253 (44%) were confirmed by the presence of a resistance-associated mutation in the *rpoB* gene, and of these, 249 had complete residential data. The majority were men (73.1%) and newly diagnosed TB cases (77.1%). The median age was 37 years (IQR 29–45), with most patients (61.9%) aged 25–44 years (table 1). Nearly half of the patients confirmed as RR-TB (47.8%) were from Kigali City. The overall RR/MDR-TB notification rate over the 8 years was 1.9 per 10 000 population, with rates across sectors ranging from 0 to 7 per 10 000 (online supplemental figure S1). Substantial spatial variation was observed in RR/MDR-TB incidence. The highest annual notification case count occurred in 2017 (n=48, 19%), followed by 2019 (n=36, 14%).

### Spatial and spatial-temporal clustering analysis of RR/MDR-TB

The global Moran’s index for RR/MDR-TB case notification rate per 10 000 population was 0.33 (p<0.01), indicating significant positive spatial autocorrelation across the study area. Kulldorff’s spatial scan statistics identified one statistically significant cluster located in Kigali City (online supplemental table S2). This cluster comprised 21 sectors mainly in Nyarugenge, southern Gasabo and western Kicukiro (online supplemental figure S2). Of all 249 patients included, 101 (40.5%) belonged to this high-prevalence cluster. The relative risk of RR/MDR-TB in this hotspot was 10.06 times higher than in the areas outside the cluster. When incorporating time into the analysis, a significant space-time cluster emerged from 2018 to 2021 in a similar area, with a relative risk of 9.39. Notably, the Kimironko sector appeared in the space-time cluster but was not part of the purely spatial cluster.

The multivariable Poisson regression analysis indicated that, at the sector level, the case notification rate of RR/MDR-TB was positively associated with population density (risk ratio: 1.09; 95% CI 1.02 to 1.15; p=0.0053) (table 2, online supplemental figure S1).

**Table 2:** Multivariate Poisson regression model of socioeconomic and demographic factors influencing the sector-level case notification rate of RR-TB per 10 000 population in Rwanda, 2017–2024

Variables	Univariate		Multivariate	
	Risk ratio (95% CI)	P value	Risk ratio (95% CI)	P value
Population density	1.18 (1.14 to 1.22)	**<0.001**	1.09 (1.02 to 1.15)	**0.0053**
Urban population (%)	9.15 (5.20 to 16.10)	**<0.001**	1.96 (0.64 to 6.05)	0.24
Gender ratio (m/f)	8.82 (3.90 to 19.93)	**<0.001**	2.63 (0.72 to 9.66)	0.14
RWI	3.97 (2.94 to 5.35)	**<0.001**	1.77 (0.91 to 3.47)	0.093
Investigation rate	1.00 (1.00 to 1.00)	0.87	–	–
Occupancy rate of correctional facilities	1.00 (0.99 to 1.01)	0.97	–	–

Statistically significant values (P value <0.05) are highlighted in bold.

RR-TB, rifampicin-resistant tuberculosis; RWI, Relative Wealth Index.

To complement this analysis, we conducted a binomial regression analysis specifically examining whether the likelihood of a sector being located within a statistically significant hotspot was associated with selected sociodemographic and economic variables. In the multivariable model, none of the variables was associated with hotspot location (online supplemental table S3).

### Molecular epidemiology

**Figure 2:**
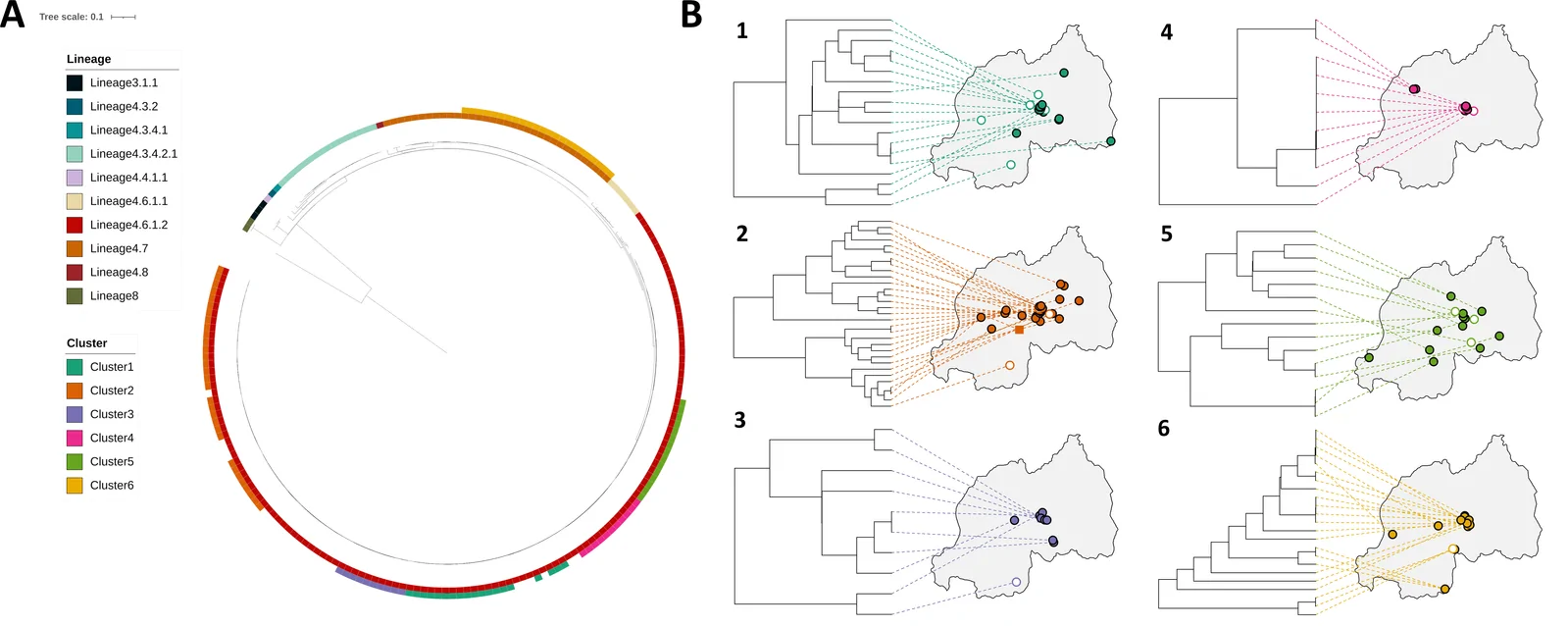
**(A**) Maximum-likelihood phylogenetic tree of the 201 isolates included in the molecular epidemiology analysis (2017–2024). The inner coloured ring indicates TB lineage, while the outer coloured ring highlights the main genotypic clusters 1–6 defined by ≤5 single-nucleotide polymorphisms. (**B**) Geographic projection of phylogenetic trees for groups 1–6, with every isolate linked with its corresponding place at the village level. Filled circles represent new RR/MDR-TB cases, white circles represent retreatment cases and rectangles represent cases with unknown treatment history. RR/MDR-TB, rifampicin resistance/multidrug-resistant tuberculosis; TB, tuberculosis.

A total of 201 RR-TB cases with residential and WGS data, excluding those with mixed-strain infection, were selected for the molecular epidemiology analysis. The majority of RR-TB cases in our cohort belong to lineage 4 (196/201, 97.5%), followed by lineage 3 (3/201, 1.5%) and lineage 8 (2/201, 1.0%) (figure 2). The majority of strains were included in clusters based on the 12-SNP cut-off (88.5%), indicating high levels of transmission in this population. With a 5-SNP threshold, 148 (73.6%) participants were allocated to genotype-specific clusters, comprising 2–34 cases, including 109 participants who belonged to six large groups of ≥10 individuals (online supplemental table S4). Five out of the six clusters were lineage 4.6.1.2, and one was lineage 4.7. Clinical characteristics were broadly similar among the clustered and non-clustered cases. Isoniazid resistance was universal among clustered cases and present in 83% (76/92) of ungrouped cases, confirming that MDR-TB strains are the predominant driver of RR-TB transmission across Rwanda.

Phylogenetic trees overlaid on a spatial map displayed heterogeneous geographic and genetic clustering patterns (figure 2). In clusters 3 and 6, most isolates appeared to aggregate in a single geographic cluster, with the majority of cases (>60%) located within a small radius (<25 km). Cluster 4 showed two distinct spatial clusters, with related isolates separated. Clusters 1, 2 and 5 had a more diffuse geographic distribution of closely related isolates.

In order to understand possible recent transmission chains, we restricted the population of cases and controls to those RR-TB cases in our dataset with no history of previous TB treatment (online supplemental table S4). To estimate spatial clustering among participants in each cluster group, we used spatial K-function analysis (online supplemental figure S3). Differences in K-functions indicated participants in clusters 3 and 4 had significantly greater spatial clustering than participants with ungrouped strains. Cluster 3 demonstrated significant positive spatial clustering at distances of 25–50 km, suggesting consistent transmission networks operating at a regional level that cross administrative boundaries. In contrast, cluster 4 exhibited intense clustering at very short distances (<15 km) followed by significant negative values beyond 50 km, indicating a concentrated urban outbreak pattern with limited geographic spread.

**Figure 3:**
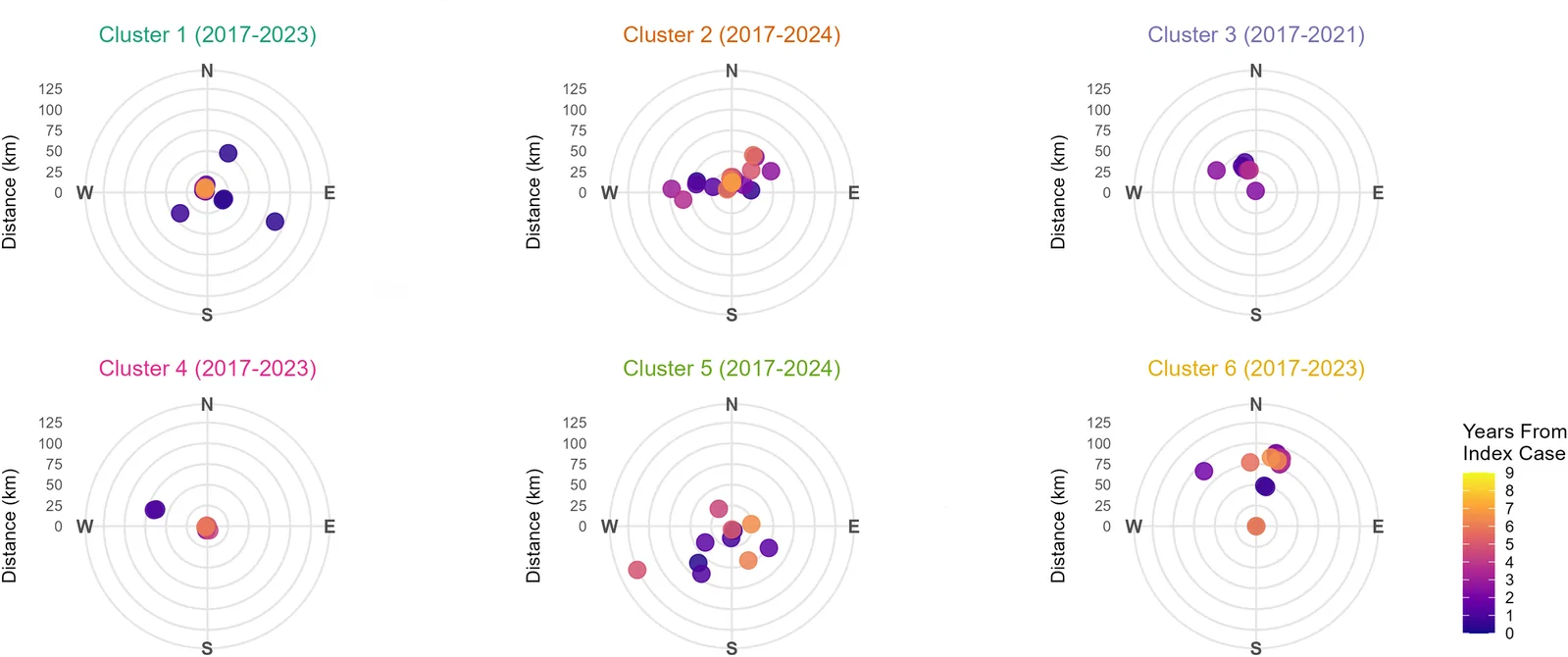
Spatial and temporal patterns, as well as the directional spread of MDR-TB cases by cluster, are shown in relation to the first diagnosed case (index case) during the study period. E, east; MDR-TB, multidrug-resistant tuberculosis; N, north; S, south; W, west.

The geographic distance between the first and subsequent cases (within our study population) diagnosed over time varied by cluster group (figure 3). For cluster 3, subsequent cases clustered around the early patient, indicating local spread. For cluster 6, subsequent cases were geographically distant from the first case but were internally tightly grouped among themselves. Cluster 4 illustrated two different spatial clusters: one near the index case, with a second emerging on a northwest axis around 1 year after this first. Cluster 1, cluster 2 and cluster 5 spread wider geographically but without a consistent pattern of increasing distance over time.

## Discussion

This study provides the first national-level spatial and molecular epidemiological assessment of MDR-TB in Rwanda. Our findings highlight significant spatial clustering of RR-TB cases within Rwanda, particularly in the capital city, with evidence of both localised and regional transmission networks that vary by strain genotype.

The significant spatial clustering of RR-TB in Kigali City, with a relative risk 10.06 times higher than areas outside the cluster, is consistent with global patterns where MDR-TB tends to concentrate in large urban environments.^40 41^ Xpert MTB/RIF Ultra has served as the first-line diagnostic tool for all presumptive patients with TB in Kigali since 2017, potentially contributing to higher case detection rates.^42^ However, our multivariable analysis revealed that the observed hotspot was not associated with the investigation rate, suggesting genuine epidemiological clustering rather than diagnostic bias. In Sub-Saharan Africa, similar urban clustering has been documented in studies from South Africa, where Cape Town was identified as a hotspot for MDR/XDR-TB transmission.^43^ These findings are consistent with previous research in Rwanda, which found that living in Kigali doubled the risk of being diagnosed with RR-TB.^6^ Kigali experiences higher outdoor air pollution levels that, combined with local meteorological conditions, compromise indoor air quality; has increased social contact rates compared with rural areas; and shows elevated prevalence of immune-compromising conditions such as diabetes and smoking.^44 45^ Future studies should measure these factors at the individual level to validate their role as drivers of RR/MDR-TB transmission.

Interestingly, our analysis revealed that the Kimironko sector (part of Kigali City province) appeared in the space-time cluster (2018–2021) but was not part of the purely spatial cluster. This discrepancy suggests that Kimironko experienced a temporally concentrated outbreak over the 3 years. Still, when analysed across the entire 8-year study period, its overall case density was insufficient to be included in the purely spatial cluster.

Our analysis examined the relationship between economic factors and RR-TB case notification rates using the RWI, where negative values represent below-average wealth and positive values represent above-average wealth. We found that RWI was positively associated with RR/MDR-TB notification rates in univariate analysis, suggesting higher case notification in wealthier-than-average areas compared with poorer-than-average areas. However, this association was attenuated in the multivariable model. This finding highlights a potential ecological fallacy, where sector-level socioeconomic measures may mask significant individual-level associations. Some studies suggest that poorer households may spend more time outdoors or live in structures with greater natural ventilation, which could potentially reduce opportunities for close-contact transmission.^46^ Research in rural Malawi reported that TB incidence may be lower than expected among poorer families as they are less likely to have glass windows, meet indoors or use enclosed public transportation.^47^ However, as we did not assess air quality or household structures in our study, the contribution of these factors remains unclear.

In contrast to previous reports, we found no association between RR-TB case notification rates and the proportion of the population living in urban areas or the proportion of men in the population.^10^ Notably, while population density was associated with RR-TB case notification rates during the study period, it did not explain the association with belonging to an RR-TB hotspot. This suggests that the primary hotspots are influenced by factors beyond crowding or detection efficiency in Kigali.

Despite prisons being well-documented hotspots for general TB transmission in Rwanda, our analysis found no significant MDR-TB clustering associated with correctional facilities. Studies have documented TB incidence in incarcerated populations exceeding 10 times that of the general population, with evidence of MDR-TB spillover from prisons to the surroundings.^48 49^ Several factors may explain this discrepancy. Rwanda conducts annual active case finding campaigns in correctional facilities among both inmates and workers, which likely contributes to early detection and treatment before MDR-TB transmission chains can be established. Additionally, MDR-TB prevalence within Rwandan prisons may genuinely differ from that of the general community, possibly reflecting differences in circulating strain composition. Taken together, this suggests that while prisons remain important sites for TB screening, they do not appear to be major drivers of MDR-TB transmission in Rwanda.

Our molecular analysis revealed that 88.5% of RR-TB cases belonged to 12-SNP clusters, indicating high levels of recent transmission. These results are consistent with earlier findings, which show that over 96% of MDR-TB cases in Rwanda result from primary transmission.^4^ Rwanda has mandatory hospitalisation for MDR-TB treatment, with patients isolated in specialised treatment centres on diagnosis.^21^ This policy likely disrupts ongoing transmission chains, which might suggest that the genomic transmission clustering we observe is primarily occurring before diagnosis or represents reactivation of latent MDR-TB infection. The persistence of genetically related clusters despite this intervention shows the challenges to interrupt transmission early enough in the infectious period. Indeed, the current testing strategies remain largely passive, relying on the reporting of symptoms. However, the 2012 national TB survey in Rwanda found that up to 40% of confirmed TB cases were asymptomatic at the time of diagnosis.^50^ Moreover, evidence suggests that transmission can occur before the onset of symptoms. A study reported that 5 out of 14 (35.7%) TB cases had likely transmitted TB well before developing symptoms, and most were sputum-negative at diagnosis.^51^

The predominance of MTB lineage 4 (97.5%) among our RR-TB cases, specifically subtype 4.6.1.2, highlights the importance of the previously described R3clone) in the national MDR-TB epidemic.^4^ This clone, resistant to all first-line drugs, was responsible for five of the six largest genotype clusters. The R3clone’s high transmissibility and established circulation within Rwanda make it a critical target for MDR-TB control efforts, as interrupting transmission of this dominant strain could substantially reduce the national burden.^4^ One study demonstrated the utility of qPCR-based detection of prevailing TB strain variants for enhanced surveillance, and similar approaches targeting the R3clone have been developed.^52^ While implementing such strain-specific molecular testing may face resource constraints in low-income settings with limited diagnostic budgets, it could be targeted to identified hotspots to maximise impact while containing costs.

By integrating genomic and spatial data, we revealed heterogeneous transmission patterns across different strain clusters. Some clusters, such as cluster 3, displayed tight spatial clustering over intermediate distances (25–50 km), which in Rwanda’s context represents substantial geographic spread that likely reflects transmission through population movement, shared transportation networks or attendance at large gatherings that bring together people from different administrative areas rather than direct local transmission. Others, like cluster 4, showed localised clustering in Kigali within 15 km and another distinct spatial cluster at different time points. This could represent either separate but related outbreak events, transmission resulting from population movement (with Kigali potentially serving as the primary transmission source and the second location representing spillover) or transmission chains linked by unsampled cases. These patterns align with previous studies showing that TB transmission is not only spatially determined but also that certain MTBC lineages tend to cluster, even after controlling for individual and socioeconomic factors.^53^ Additionally, these findings challenge the notion that RR-TB strains are less transmissible.^54^

Importantly, all clusters showed transmission extending beyond Kigali City, with five clusters (1–3, 5 and 6) having only 40%–65% of their isolates located in Kigali province. This highlights the need not only to perform spatial analysis but also to examine transmission dynamics, which may reveal previously undetected transmission patterns.^14 16^

In Rwanda, prior work has shown that household contacts of patients with RR-TB have an 11-fold increased risk of infection.^6^ Patients treated for RR/MDR-TB are recommended to invite their household contacts for investigation in the TB diagnostic centres, with variable uptake. Household contact screening remains important, especially for those eligible for TB preventive therapy.^55^ However, at a population level, our findings indicate that substantial non-household transmission occurs, suggesting contact investigation should go beyond co-habitants to include patients’ broader social and activity networks. Molecular epidemiology studies in rural Uganda have identified drinking venues and healthcare settings as high-risk locations for TB transmission, with genotype-clustered cases often not sharing households.^56^ The identification of distinct spatial clusters with genetically related strains suggests that activity spaces, geographic areas where people spend time during regular daily activities, may be important sites for MDR-TB transmission.^18^ In Rwanda, such activities may include gatherings in poorly ventilated spaces, such as house-based prayer meetings, churches, markets, public transportation and confined workplaces like mining facilities.

The significant urban clustering in Kigali suggests that intensified case-finding efforts in identified hotspots could yield high returns. While Rwanda’s National TB Programme currently conducts active case finding in prisons, youth transit centres and some TB hotspots/slums in Kigali using symptom screening and chest X-ray, these approaches have primarily focused on general TB detection rather than specifically targeting MDR-TB cases.^57^ This may explain the limited yield of confirmed MDR-TB cases from these interventions, as the epidemiological context and frequency of general TB do not always align with that of RR/MDR-TB. Our study did not cover drug-susceptible TB; hence, further comparative studies examining both drug-susceptible and drug-resistant strains would be needed to explore potential differences in transmission frequency and patterns.

This study has several strengths, including its national scope, integration of molecular and spatial data and use of advanced analytical methods to assess transmission patterns. The high proportion of total cases (74%) included in our analysis relative to WHO notifications provides confidence in the representativeness of our findings.

However, several limitations must be acknowledged. First, our ecological analysis lacks granular prevalence estimates of key factors associated with DR-TB (ie, air pollution, nutritional status, alcohol dependence, smoking, diabetes), which hinders ecological analyses. Second, the isolates included in the analyses do not represent all RR-TB cases in Rwanda over the 8-year study period, as some cases may not have been diagnosed. Moreover, we were unable to obtain *rpoB* sequencing data for all initially detected cases due to culture failures, contamination or insufficient DNA yield. Additionally, we lacked precise residential information for a small portion of confirmed RR-TB cases. Third, the identification of MDR-TB clusters was based on genotype clustering, which does not definitively prove direct person-to-person transmission without additional epidemiological data (e.g. occupations, places of employment, common social gathering sites).^58^ Finally, although we included both actively and passively detected cases, the balance between these sampling approaches may have varied across regions and over time, potentially influencing the observed spatial patterns.

## Conclusion

This study is the first to provide critical insight into the spatial and molecular dynamics of MDR-TB in Rwanda. The identification of widespread genotype-linked transmission points to the need for targeted and expanded community-level case finding and molecular surveillance. Household tracing, while essential, is insufficient to capture the whole transmission network of MDR-TB. Future studies should investigate the social and environmental contexts that drive the transmission of genetically related strains across seemingly disconnected geographic areas. This integrated approach could support more targeted, evidence-based interventions in Rwanda that target actual transmission networks rather than presumed geographic hotspots.

## Supplementary material

10.1136/bmjopen-2025-111823 online supplemental file 1

## Data Availability

All data relevant to the study are included in the article or uploaded as supplementary information.

## References

[1] WHO (2024). Global tuberculosis report. https://www.who.int/publications/i/item/9789240101531.

[2] Kendall EA, Fofana MO, Dowdy DW (2015). Burden of transmitted multidrug resistance in epidemics of tuberculosis: a transmission modelling analysis. Lancet Respir Med.

[3] Dheda K, Gumbo T, Maartens G (2017). The epidemiology, pathogenesis, transmission, diagnosis, and management of multidrug-resistant, extensively drug-resistant, and incurable tuberculosis. Lancet Respir Med.

[4] Ngabonziza JCS, Rigouts L, Torrea G (2022). Multidrug-resistant tuberculosis control in Rwanda overcomes a successful clone that causes most disease over a quarter century. J Clin Tuberc Other Mycobact Dis.

[5] Dowdy DW, Golub JE, Chaisson RE (2012). Heterogeneity in tuberculosis transmission and the role of geographic hotspots in propagating epidemics. *Proc Natl Acad Sci U S A*.

[6] Habimana-Mucyo Y, Dushime A, Migambi P (2023). Continuous surveillance of drug-resistant TB burden in Rwanda: a retrospective cross-sectional study. Int Health.

[7] Cuella-Martin I, Hakizayezu F, Ahmed A (2025). Paucibacillary Tuberculosis Drives the Low Positive Predictive Value of Xpert MTB/RIF Ultra for Rifampicin Resistance Detection in Low-Prevalence Settings. Clin Infect Dis.

[8] Swindells S, Gupta A, Kim S (2018). Resource utilization for multidrug-resistant tuberculosis household contact investigations (A5300/I2003). *int j tuberc lung dis*.

[9] Manjourides J, Lin H-H, Shin S (2012). Identifying multidrug resistant tuberculosis transmission hotspots using routinely collected data. *Tuberculosis (Edinb*).

[10] Alene KA, Viney K, McBryde ES (2017). Spatial patterns of multidrug resistant tuberculosis and relationships to socio-economic, demographic and household factors in northwest Ethiopia. *PLoS One*.

[11] Soto A (2024). Prisons as boosters of tuberculosis and drug resistance tuberculosis transmission in Latin America. Lancet Reg Health Am.

[12] Yang C, Lu L, Warren JL (2018). Internal migration and transmission dynamics of tuberculosis in Shanghai, China: an epidemiological, spatial, genomic analysis. Lancet Infect Dis.

[13] Najafizada M, Rahman A, Taufique Q (2021). Social determinants of multidrug-resistant tuberculosis: A scoping review and research gaps. Indian J Tuberc.

[14] Cudahy PGT, Andrews JR, Bilinski A (2019). Spatially targeted screening to reduce tuberculosis transmission in high-incidence settings. Lancet Infect Dis.

[15] Lan Y, Crudu V, Ciobanu N (2024). Identifying local foci of tuberculosis transmission in Moldova using a spatial multinomial logistic regression model. *EBioMedicine*.

[16] Zelner JL, Murray MB, Becerra MC (2016). Identifying Hotspots of Multidrug-Resistant Tuberculosis Transmission Using Spatial and Molecular Genetic Data. J Infect Dis.

[17] Baker CR, Barilar I, de Araujo LS (2023). Use of High-Resolution Geospatial and Genomic Data to Characterize Recent Tuberculosis Transmission, Botswana. *Emerg Infect Dis*.

[18] Bui DP, Chandran SS, Oren E (2021). Community transmission of multidrug-resistant tuberculosis is associated with activity space overlap in Lima, Peru. BMC Infect Dis.

[19] (2025). Fifth population and housing census - 2022. https://www.statistics.gov.rw/datasource/fifth-population-and-housing-census-2022.

[20] (2025). RPHC5 thematic report:population size, structure and distribution. https://statistics.gov.rw/publication/1975.

[21] (2024). Tuberculosis and other respiratory communicable diseases division annual report 2023-2024.

[22] Ngabonziza JCS, Decroo T, Migambi P (2020). Prevalence and drivers of false-positive rifampicin-resistant Xpert MTB/RIF results: a prospective observational study in Rwanda. *The Lancet Microbe*.

[23] Rigouts L, Nolasco O, de Rijk P (2007). Newly developed primers for comprehensive amplification of the rpoB gene and detection of rifampin resistance in Mycobacterium tuberculosis. J Clin Microbiol.

[24] Kulldorff M, Nagarwalla N (1995). Spatial disease clusters: Detection and inference. Stat Med.

[25] (2025). R: a language and environment for statistical computing. https://www.gbif.org/tool/81287/r-a-language-and-environment-for-statistical-computing.

[26] QGIS Development Team (2022). QGIS geographic information system. Open source geospatial foundation. http://qgis.org.

[27] Kohl TA, Utpatel C, Schleusener V (2018). MTBseq: a comprehensive pipeline for whole genome sequence analysis of *Mycobacterium tuberculosis* complex isolates. PeerJ.

[28] Kozlov AM, Darriba D, Flouri T (2019). RAxML-NG: a fast, scalable and user-friendly tool for maximum likelihood phylogenetic inference. Bioinformatics.

[29] Leaché AD, Banbury BL, Felsenstein J (2015). Short Tree, Long Tree, Right Tree, Wrong Tree: New Acquisition Bias Corrections for Inferring SNP Phylogenies. Syst Biol.

[30] Stewart Fotheringham A, Rogerson AP, Waller LA (2009). The SAGE handbook of spatial analysis.

[31] Wickham H, Chang W, Henry L (2025). ggplot2: create elegant data visualisations using the grammar of graphics. https://cran.r-project.org/web/packages/ggplot2/index.html.

[32] Baddeley A, Turner R, Rubak E (2025). spatstat: spatial point pattern analysis, model-fitting, simulation, tests. https://cran.r-project.org/web/packages/spatstat/index.html.

[33] Pebesma E, Bivand R, Rowlingson B (2025). sp: classes and methods for spatial data. https://cran.r-project.org/web/packages/sp/index.html.

[34] Pebesma E, Bivand R, Racine E (2025). sf: simple features for R. https://cran.r-project.org/web/packages/sf/index.html.

[35] Hijmans RJ, Williams E, Vennes C (2024). geosphere: spherical trigonometry. https://cran.r-project.org/web/packages/geosphere/index.html.

[36] Paradis E, Blomberg S, Bolker B (2024). ape: analyses of phylogenetics and evolution. https://cran.r-project.org/web/packages/ape/index.html.

[37] Revell LJ (2025). phytools: phylogenetic tools for comparative biology (and other things). https://cran.r-project.org/web/packages/phytools/index.html.

[38] (2022). Brownrigg OS code by RAB and ARWR version by R. mapdata: extra map databases. https://cran.r-project.org/web/packages/mapdata/index.html.

[39] Letunic I, Bork P (2007). Interactive Tree Of Life (iTOL): an online tool for phylogenetic tree display and annotation. Bioinformatics.

[40] Jenkins HE, Gegia M, Furin J (2014). Geographical heterogeneity of multidrug-resistant tuberculosis in Georgia, January 2009 to June 2011. Eurosurveillance.

[41] Wang Z, Hou Y, Guo T (2023). Epidemiological characteristics and risk factors of multidrug-resistant tuberculosis in Luoyang, China. Front Public Health.

[42] Ngabonziza J-CS, Habimana YM, Decroo T (2020). Reduction of diagnostic and treatment delays reduces rifampicin-resistant tuberculosis mortality in Rwanda. Int J Tuberc Lung Dis.

[43] Oostvogels S, Ley SD, Heupink TH (2022). Transmission, distribution and drug resistance-conferring mutations of extensively drug-resistant tuberculosis in the Western Cape Province, South Africa. Microb Genom.

[44] Nibagwire D, Ana G, Kalisa E (2025). Analysis of the influence of exogenous factors on indoor air quality in residential buildings. Front Built Environ.

[45] NCD Division (2022). The second Rwanda non-communicable diseases risk factors study. https://rbc.gov.rw/rnhrr/article?code=186.

[46] Chamie G, Wandera B, Luetkemeyer A (2013). Household ventilation and tuberculosis transmission in Kampala, Uganda. Int J Tuberc Lung Dis.

[47] Odone A, Crampin AC, Mwinuka V (2013). Association between Socioeconomic Position and Tuberculosis in a Large Population-Based Study in Rural Malawi. PLOS ONE.

[48] Walter KS, Dos Santos PCP, Gonçalves TO (2022). The role of prisons in disseminating tuberculosis in Brazil: A genomic epidemiology study. Lancet Reg Health Am.

[49] Warren JL, Grandjean L, Moore DAJ (2018). Investigating spillover of multidrug-resistant tuberculosis from a prison: a spatial and molecular epidemiological analysis. BMC Med.

[50] Migambi P, Gasana M, Uwizeye CB (2020). Prevalence of tuberculosis in Rwanda: Results of the first nationwide survey in 2012 yielded important lessons for TB control. PLOS ONE.

[51] Xu Y, Cancino-Muñoz I, Torres-Puente M (2019). High-resolution mapping of tuberculosis transmission: Whole genome sequencing and phylogenetic modelling of a cohort from Valencia Region, Spain. PLoS Med.

[52] Pérez-Lago L, Izco S, Herranz M (2017). A novel strategy based on genomics and specific PCR reveals how a multidrug-resistant Mycobacterium tuberculosis strain became prevalent in Equatorial Guinea 15 years after its emergence. Clin Microbiol Infect.

[53] Ribeiro FKC, Pan W, Bertolde A (2015). Genotypic and Spatial Analysis of Mycobacterium tuberculosis Transmission in a High-Incidence Urban Setting. *Clin Infect Dis*.

[54] de Vos M, Müller B, Borrell S (2013). Putative compensatory mutations in the rpoC gene of rifampin-resistant Mycobacterium tuberculosis are associated with ongoing transmission. Antimicrob Agents Chemother.

[55] Mathema B, Andrews JR, Cohen T (2017). Drivers of Tuberculosis Transmission. J Infect Dis.

[56] Chamie G, Wandera B, Marquez C (2015). Identifying locations of recent TB transmission in rural Uganda: a multidisciplinary approach. Trop Med Int Health.

[57] (2022). Tuberculosis and other respiratory communicable diseases division annual report 2021-2022.

[58] Devaux I, Kremer K, Heersma H (2009). Clusters of multidrug-resistant Mycobacterium tuberculosis cases, Europe. Emerg Infect Dis.

