# Supplementary material for: Spatial clustering and transmission networks of multidrug-resistant tuberculosis in Rwanda: a national retrospective genomic and spatial epidemiological study
Source: BMJ Open. 2026 Aug 26;16(8):e111823. doi: 10.1136/bmjopen-2025-111823 (PMC13536106; doi:10.1136/bmjopen-2025-111823)
Supplement: Supplementary file 1 [file bmjopen-2025-111823.reviewer_comments.pdf]

PEER REVIEW HISTORY

BMJ Open publishes all reviews undertaken for accepted manuscripts. Reviewers are asked to complete a checklist review form and are provided with free text boxes to elaborate on their assessment. These free text comments are reproduced below.

ARTICLE DETAILS

Title (Provisional)

Spatial clustering and transmission networks of multidrug-resistant tuberculosis in Rwanda: a national retrospective genomic and spatial epidemiological study

Authors

Cuella-Martin, Isabel; Hakizayezu, Francois; Mulders, Wim; Niyompano, Hosee; Runyambo, Docteur; Keysers, Jelle; De Rijk, Willem B; HABIMANA MUCYO, Yves; Migambi, Patrick; Muvunyi, CM; Meehan, Conor J; de Jong, Bouke C; Rigouts, Leen; Ngabonziza, JCS; Mitchell, Ellen

---

VERSION 1 - REVIEW

Reviewer **1**

Name **Xu, Lin**

Affiliation **Yunnan Center for Disease Control and Prevention, Division of tuberculosis control and prevention**

Date **26-Oct-2025**

COI **None**

This study is very interesting and valuable. The study design and methods are very clear and the results are very reliable. It is original, timely and highly relevant to elimination policies. However, there are some points needs to be explain.

1. How about WGS quality control? please provide much more details because it is the most important part for whole study results.

2. In results, 164/421 Xpert “RR” cases carried wild-type rpoB and were excluded. Why and how much influence for the results of this study?

Reviewer **2**

Name **Ruswa, Nunurai**

Affiliation **National Tuberculosis and Leprosy Control Programme**

Date **05-Jan-2026**

**COI**	**None**

I congratulate the authors for submitting this novel article on Integrating spatial epidemiology with genomic analysis for multi-drug resistant TB in Rwanda and thank them for the opportunity to review it.

The manuscript is well-written with only minor issues for revision noted. These are outlined below:

Results

Of the 573 RR/MDR cases in the period under review, only 201 RR-TB cases had residential and WGS data. The authors allude to performing sensitivity analysis in their Methods section. However, there is no mention of the results of the sensitivity analysis or potential selection bias resulting from the drop from 573 to 201. The authors are encouraged to discuss the results of the sensitivity analysis, add any assessment of the RR/MDR cases that were excluded, discuss the inability to do so as a limitation, and/or add an appropriate table in the supplementary materials.

Discussion:

The authors suggest a potential ecological fallacy when explaining the higher RR/MDR case-notification rates in wealthier areas. They, however, may need to discuss the more obvious role of the availability of diagnostic services in wealthier areas, which may drive notification rates up.

Supplementary materials

Table S1 lists the proportions of Cases with confirmed rpoB mutation associated with resistance per year. These vary significantly, from 75% in 2017 to 88% in 2020, 39% in 2023 and 70% in 2024. The potential causes and/or implications of this should be alluded to somewhere in the article.

**Reviewer**	**3**
**Name**	**Lin, Hsien-Ho**
**Affiliation**	**National Taiwan University**
**Date**	**19-Mar-2026**
**COI**	**None**

The study presents a valuable integration of genomic epidemiology and spatial analysis to characterize MDR-TB transmission in Rwanda. The combined use of molecular and spatial data provides important insights into transmission dynamics and has clear relevance for public health interventions. However, several aspects of the manuscript would benefit from

further clarification to improve readability and ensure that key methods and findings are clearly interpreted by the reader.

Comments

Abstract

“Molecular analysis revealed 88.5% of cases belonged to genotype clusters, with 73.6% forming clusters at a strict 5-SNP threshold.”

Not clear what this means. Does it mean that the 88.5% clustering was defined based on a less strict threshold?

Methods

1. In study setting and population, the last sentence: “Targeted

population groups, such as inmates and the elderly, are disproportionately affected, together … accounting for 56.5% of all TB cases in fiscal year 2023-2024”.

It is unclear what is meant by “targeted population groups”.

Additionally, this sentence refers to the contribution to all TB cases, whereas, the paragraph appears to discussing risk factors or epidemiology of MDR-TB/RR-TB?

2. p.5 Line 47: “During this period, the GeneXpert MTB/RIF (Xpert) and GeneXpert MTB/RIF Ultra (Ultra) assays (Cepheid, Sunnyvale, CA, USA) were used as first-line diagnostic tools for high-risk and priority groups.”

Please clarify which specific groups are included in the high-risk and priority groups.

“Additionally, GeneXpert was used nationwide to detect RIF resistance in patients

with microscopically diagnosed TB:”

It would be helpful to clarify the diagnostic pathway for RR-TB detection. For example: Were all smear-positive TB cases subsequently tested using GeneXpert for rifampicin resistance? Were smear-negative cases also tested with GeneXpert?

What proportion of diagnosed TB cases underwent GeneXpert testing?

Providing a short description or flow of the diagnostic algorithm would improve clarity. The recruitment process of patients included in the RR/MDR-TB clinic is not entirely clear. For example, among individuals diagnosed with RR/MDR-TB, what proportion were successfully admitted to the RR/MDR clinic? Were all patients interviewed and sampled for sputum testing? Related numbers at Table S1 but maybe include some information in the main text

3. Related to the previous comment. Please comment on the coverage of the study samples among all potential RR cases in Rwanda, and the implication of the coverage on the study results and conclusion. For example, could potential transmission clusters be missed due to incomplete sampling?

4. p.7 Line 41, how was mixed-strain infection defined?

5. p.7 “Analysis focused on six clusters (>=10 individuals with a shared genotype), with ungrouped cases (those not belonging to any cluster >=10 individuals) as a comparison. “ It appears that the analysis compares cases belonging to large clusters (≥10 individuals) with those not belonging to such clusters. Please clarify the rationale for selecting this comparison group. In particular, the interpretation of the analysis may reflect transmission within large clusters compared with smaller clusters or non-clustered cases.

6. The description of the statistical analysis is somewhat difficult to follow. In particular, the spatial analyses are presented across multiple sections, which makes it challenging to understand the overall analytical framework. It would be helpful to consolidate these descriptions or provide a clearer structure outlining the sequence of analyses.

In addition, a relevant reference for the K-function analysis should be included.

Results

7. Regarding “573 cases of RR/MDR-TB diagnosed using Xpert MTB/RIF, 253 were confirmed by the presence of rpoB gene.” It would be helpful to clarify the remaining 56% were negative for rpoB mutations or not being tested.

8. Are the 201 RR-TB cases in the molecular epidemiology analysis different from those not included in the genomic analysis?

9. p.9 “The multivariable Poisson regression analysis indicated that, at the sector level, the case notification rate of RR/MDR-TB was positively associated with population density (risk ratio: 1.00; 95% CI: 1.00–1.00; P=0.0053)” Although statistically significant, does the risk ratio of 1.00 mean positively associated? Please consider to clarify the unit used in the model and consider presenting the effect estimate using a more interpretable scale.

10. “All cases inside clusters were resistant to isoniazid, showing that MDR-TB strains drive the ongoing transmission of RR-TB” This interpretation may be somewhat overstated based on the presented results. Table S4 showed that a large proportion of ungrouped cases were also MDR-TB.

11. p.9 line 46-47, please clarify how “aggregate in a single geographic cluster” was defined.

12. Figure 2A. The phylogenetic tree was barely visible.

13. Figure S1A. There were 573 (?) notified RR cases over the 416 sectors. How stable was the spatial pattern given that each sector would end up with very few (or even zero) cases.

Discussion

14. “Despite prisons being well-documented hotspots for general TB transmission in Rwanda, our analysis found no significant MDR-TB clustering associated with correctional facilities”. If general TB transmission is higher in prisons but MDR-TB clustering is not

observed, it would be helpful to discuss possible explanations. Could MDR-TB prevalence be lower within prisons compared with the general community?

Could this result reflect limited statistical power or incomplete sampling?

15. p.13 Line 8 “Importantly, all clusters showed transmission extending beyond Kigali City, with five clusters (1-3, 5, and 6) having only 40-65% of their isolates originating from Kigali province. This highlights the need not only to perform spatial analysis but also to examine transmission dynamics, which may uncover previously undetected transmission patterns.” While this observation is interesting, the interpretation that transmission originated from the first notified case should be made cautiously. Without a formal transmission tree reconstruction, the first notified case in a cluster may not represent the true index case especially in tuberculosis transmission.

16. Regarding the discussion of household contact, are there any data showing the proportion of clustered cases with documented household contact? Or if epidemiological investigation data were unavailable, were there cases sharing identical residential addresses? It might make the discussion more evidence-based.

VERSION 1 - AUTHOR RESPONSE

Reviewer: 1

Dr. Lin Xu, Yunnan Center for Disease Control and Prevention

Comments to the Author:

This study is very interesting and valuable. The study design and methods are very clear and the results are very reliable. It is original, timely and highly relevant to elimination policies. However, there are some points needs to be explain.

1. How about WGS quality control? please provide much more details because it is the most important part for whole study results.

We agree that WGS quality control is a critical component of the analysis. As this methodology has been described in detail in previously published work from our group (references [7] and [22]), we directed readers to these publications rather than repeating the full pipeline in the current manuscript. Briefly, non-MTBC reads were filtered from FASTQ datasets using Centrifuge v1.0.3, retaining only reads mapping to any MTBC variant. Samples with less than 95% of reads mapped to the MTBC were excluded. Reads were then mapped to the inferred ancestral M. tuberculosis genome using MTBseq with default values, with a variant filter set at 5%. WGS-based resistance profiles were defined using TB-Profiler v2.6 with the tbdb mutation database. Isolates without a known RR-conferring mutation, those showing polyclonal TB infection, or those showing heteroresistance (mutant population <95%) were excluded from further analysis.

2. In results, 164/421 Xpert “RR” cases carried wild-type rpoB and were excluded. Why and how much influence for the results of this study?

The 164 wild-type rpoB cases represent false-positive rifampicin resistance calls by GeneXpert, a phenomenon that has been described in detail in our previous publications (reference 7 and 22). As these cases do not carry a confirmed resistance-associated mutation, their exclusion is methodologically necessary, including them would introduce misclassification bias by incorporating non-

RR-TB cases into an analysis of RR/MDR-TB transmission dynamics. The influence on the results of the current study is therefore not a concern of selection bias but rather of diagnostic accuracy, and their exclusion strengthens rather than undermines the validity of the findings

Reviewer: 2

Dr. Nunurai Ruswa, National Tuberculosis and Leprosy Control Programme

Comments to the Author:

I congratulate the authors for submitting this novel article on Integrating spatial epidemiology with genomic analysis for multi-drug resistant TB in Rwanda and thank them for the opportunity to review it. The manuscript is well-written with only minor issues for revision noted. These are outlined below:

Results

Of the 573 RR/MDR cases in the period under review, only 201 RR-TB cases had residential and WGS data. The authors allude to performing sensitivity analysis in their Methods section. However, there is no mention of the results of the sensitivity analysis or potential selection bias resulting from the drop from 573 to 201. The authors are encouraged to discuss the results of the sensitivity analysis, add any assessment of the RR/MDR cases that were excluded, discuss the inability to do so as a limitation, and/or add an appropriate table in the supplementary materials.

The broader drop from 573 notified to 249 cases is largely explained by diagnostic issues, primarily false-positive Xpert results (n=164), poor quality WGS, (n=4) and culture failures (n=152) rather than systematic exclusion. A comparison of demographic and clinical characteristics between 249 confirmed RR-TB cases with WGS-available (n=201) and WGS-not available (n=48) cases revealed no statistically significant differences, suggesting no systematic selection bias in the molecular epidemiology analysis (Table 1).

The inability to obtain WGS data for all confirmed cases has been acknowledged in the limitations section.

We want to clarify that the "sensitivity analyses" referred to in the methods section relate to the complementary regression analysis conducted to validate that identified hotspots reflected genuine epidemiological clustering rather than detection bias.

Discussion:

The authors suggest a potential ecological fallacy when explaining the higher RR/MDR case-notification rates in wealthier areas. They, however, may need to discuss the more obvious role of the availability of diagnostic services in wealthier areas, which may drive notification rates up.

We agree that the availability of diagnostic services in wealthier areas is an important potential confounder of the observed association between RWI and case notification rates. However, this was explicitly accounted for in our multivariable model through the inclusion of the investigation rate as a covariate, which captures the volume of GeneXpert and smear microscopy testing performed at the district level. The investigation rate was not significantly associated with case notification rates in either the univariate or multivariable analysis, suggesting that differential diagnostic access does not explain the observed spatial patterns.

Supplementary materials

Table S1 lists the proportions of Cases with confirmed rpoB mutation associated with resistance per year. These vary significantly, from 75% in 2017 to 88% in 2020, 39% in 2023 and 70% in 2024. The potential causes and/or implications of this should be alluded to somewhere in the article.

The variation in annual confirmation rates reflects evolving diagnostic algorithms and testing practices over the study period, including the adjustments to GeneXpert testing protocols following the recognition of its higher false-positive rate for RIF resistance in paucibacillary samples, and the subsequent progressive rollout of Xpert Ultra, as described in detail in references 7 and 22. These diagnostic changes and their implications are already briefly discussed in the manuscript.

Reviewer: 3

Dr. Hsien-Ho Lin, National Taiwan University

Comments to the Author:

The study presents a valuable integration of genomic epidemiology and spatial analysis to characterize MDR-TB transmission in Rwanda. The combined use of molecular and spatial data provides important insights into transmission dynamics and has clear relevance for public health interventions. However, several aspects of the manuscript would benefit from further clarification to improve readability and ensure that key methods and findings are clearly interpreted by the reader.

Comments

Abstract

“Molecular analysis revealed 88.5% of cases belonged to genotype clusters, with 73.6% forming clusters at a strict 5-SNP threshold.” Not clear what this means. Does it mean that the 88.5% clustering was defined based on a less strict threshold?

We agree that the sentence was ambiguous. We have clarified that the 88.5% figure refers to a 12-SNP threshold, while the 73.6% refers to the stricter 5-SNP threshold.

Methods

1. In study setting and population, the last sentence: “Targeted population groups, such as inmates and the elderly, are disproportionately affected, together … accounting for 56.5% of all TB cases in fiscal year 2023-2024”. It is unclear what is meant by “targeted population groups”. Additionally, this sentence refers to the contribution to all TB cases, whereas, the paragraph appears to discussing risk factors or epidemiology of MDR-TB/RR-TB?

You are correct that this sentence referred to all TB cases rather than MDR-TB/RR-TB specifically. We have removed it to avoid confusion.

2. p.5 Line 47: “During this period, the GeneXpert MTB/RIF (Xpert) and GeneXpert MTB/RIF Ultra (Ultra) assays (Cepheid, Sunnyvale, CA, USA) were used as first-line diagnostic tools for high-risk and priority groups.” Please clarify which specific groups are included in the high-risk and priority groups.

“Additionally, GeneXpert was used nationwide to detect RIF resistance in patients with microscopically diagnosed TB:” It would be helpful to clarify the diagnostic pathway for RR-TB detection. For example: Were all smear-positive TB cases subsequently tested using GeneXpert for rifampicin resistance? Were smear-negative cases also tested with GeneXpert? What proportion of diagnosed TB cases underwent GeneXpert testing.

Providing a short description or flow of the diagnostic algorithm would improve clarity. The recruitment process of patients included in the RR/MDR-TB clinic is not entirely clear. For example, among individuals diagnosed with RR/MDR-TB, what proportion were successfully admitted to the RR/MDR clinic? Were all patients interviewed and sampled for sputum testing? Related numbers at Table S1 but maybe include some information in the main text

We have clarified the diagnostic algorithm in Methods, specifying the role of the GeneXpert platform both in Kigali and outside Kigali, and the specific high-risk and priority groups. We have also clarified that all patients diagnosed with RR/MDR-TB are mandatorily admitted to the RR/MDR-TB clinic, where they are enrolled into the study and sampled for sputum testing. Importantly, as mentioned in the Introduction section, Rwanda has achieved high coverage of drug-susceptibility testing, with 96% of new TB cases and 94% of previously treated cases undergoing RR testing.

3. Related to the previous comment. Please comment on the coverage of the study samples among all potential RR cases in Rwanda, and the implication of the coverage on the study results and conclusion. For example, could potential transmission clusters be missed due to incomplete sampling?

This is acknowledged as a limitation in the strengths and limitations section of the revised manuscript. The potential role of unsampled cases is already noted in the discussion.

4. p.7 Line 41, how was mixed-strain infection defined?

We have added a brief definition of mixed-strain infection in the Methods section: "defined as polyclonal TB infection (i.e. multiple strains in one sample) or heteroresistance (i.e. mixed population of drug-resistant mutants and wildtype, when the mutant population is still < 95%)".

5. p.7 "Analysis focused on six clusters (>=10 individuals with a shared genotype), with ungrouped cases (those not belonging to any cluster >=10 individuals) as a comparison. " It appears that the analysis compares cases belonging to large clusters (≥10 individuals) with those not belonging to such clusters. Please clarify the rationale for selecting this comparison group. In particular, the interpretation of the analysis may reflect transmission within large clusters compared with smaller clusters or non-clustered cases.

The threshold of ≥10 individuals was selected pragmatically to ensure sufficient sample sizes for meaningful spatial K-function analysis, and is consistent with definitions used in the literature to identify outbreak groups (Baker *et al*, 2023).

The comparison group of "ungrouped cases", those not belonging to any cluster of equal or more than 10 individuals, was included to represent the underlying density of RR/MDR-TB infection in the population, rather than as a direct comparator to large clusters. We acknowledge that this distinction was not sufficiently clear in the original text and have clarified it accordingly.

6. The description of the statistical analysis is somewhat difficult to follow. In particular, the spatial analyses are presented across multiple sections, which makes it challenging to understand the overall analytical framework. It would be helpful to consolidate these descriptions or provide a clearer structure outlining the sequence of analyses. In addition, a relevant reference for the K-function analysis should be included.

We agree that the spatial analyses are spread across multiple sections, which can make the overall analytical framework difficult to follow. This structure was intentional, as we organised the methods to mirror the sequence of the results section, separating the ecological spatial analysis from the molecular transmission analysis. To improve clarity, subtitles have been matched accordingly, and we have added a brief introductory sentence at the start of the sections outlining the overall analytical sequence.

The relevant reference for the K-function analysis has also been added (ref 30).

Results

7. Regarding "573 cases of RR/MDR-TB diagnosed using Xpert MTB/RIF, 253 were confirmed by the presence of rpoB gene." It would be helpful to clarify the remaining 56% were negative for rpoB mutations or not being tested.

We clarify that of the 573 nationally notified RR/MDR-TB cases, 421 underwent rpoB sequencing, with the remainder not tested primarily due to culture failures or insufficient sample quality. Of the 421 tested, 253 were confirmed as RR by the presence of an rpoB resistance-associated mutation, 164 were rpoB wild-type (consistent with false-positive Xpert results, as described previously by our group in references 7 and 22), and 4 had poor sequence quality. This breakdown is fully illustrated in Figure 1.

8. Are the 201 RR-TB cases in the molecular epidemiology analysis different from those not included in the genomic analysis?

To assess potential selection bias resulting from the drop from 249 cases to 201 cases with both residential and WGS data, we compared the demographic and clinical characteristics of WGS-available(n=201) and WGS-not available (n=48) cases from the spatial analysis dataset (n=249). No statistically significant differences were found between the two groups for any variable examined. This comparison has been added as an expanded Table 1 in the manuscript and updated in the method section.

9. p.9 “The multivariable Poisson regression analysis indicated that, at the sector level, the case notification rate of RR/MDR-TB was positively associated with population density (risk ratio: 1.00; 95% CI: 1.00–1.00; P=0.0053)” Although statistically significant, does the risk ratio of 1.00 mean positively associated? Please consider to clarify the unit used in the model and consider presenting the effect estimate using a more interpretable scale.

Presenting the effect estimate per unit (1 person/km²) results in a risk ratio of 1.00, which is hardly interpretable. We have rescaled population density to per 1,000 people/km² in the model, which produces a more meaningful effect estimate while leaving the statistical inference unchanged. The updated risk ratio indicates that each increase of 1,000 people/km² is associated with an 8.6% increase in the sector-level RR/MDR-TB case notification rate. We have updated the text (Methods and Results) and Table 2 accordingly.

10. “All cases inside clusters were resistant to isoniazid, showing that MDR-TB strains drive the ongoing transmission of RR-TB” This interpretation may be somewhat overstated based on the presented results. Table S4 showed that a large proportion of ungrouped cases were also MDR-TB.

The original statement remains valid, as all clustered cases were isoniazid-resistant. However, we have revised the sentence to incorporate the isoniazid resistance data from ungrouped cases, which further strengthens this conclusion.

11. p.9 line 46-47, please clarify how “aggregate in a single geographic cluster” was defined

The term "aggregate in a single geographic cluster" refers to a visual assessment from Figure 2B, supported by the geographic distance data, which shows that the majority of isolates (more than 60%) in these clusters originated from locations in a radius smaller than 25km. We have clarified this in the text.

12. Figure 2A. The phylogenetic tree was barely visible.

Figure 2A has been provided in a higher resolution formatted file suitable for journal production, which should ensure optimal visibility upon publication.

13. Figure S1A. There were 573 (?) notified RR cases over the 416 sectors. How stable was the spatial pattern given that each sector would end up with very few (or even zero) cases.

We acknowledge that a substantial number of sectors recorded zero cases during the study period. However, several considerations support the stability and validity of the observed spatial patterns. First, notification rates were calculated over the entire 8-year study period rather than annually, which maximises case accumulation per sector and improves rate stability. Second, to account for potential detection bias from differential test access across sectors, the investigation rate was included as a

covariate in the regression analysis, and was not significantly associated with case notification rates, suggesting that the observed spatial patterns reflect genuine epidemiological clustering rather than detection artefacts. Finally, the spatial clustering analysis using SaTScan is specifically designed to handle sparse count data by using Monte Carlo permutation testing, making it robust to the presence of zero-case areas.

Discussion

14. "Despite prisons being well-documented hotspots for general TB transmission in Rwanda, our analysis found no significant MDR-TB clustering associated with correctional facilities". If general TB transmission is higher in prisons but MDR-TB clustering is not observed, it would be helpful to discuss possible explanations. Could MDR-TB prevalence be lower within prisons compared with the general community? Could this result reflect limited statistical power or incomplete sampling?

We appreciate this comment and agree that the absence of MDR-TB clustering around correctional facilities warrants further discussion. Several explanations may account for this finding. First, while prisons are well-documented hotspots for drug-susceptible TB transmission, MDR-TB prevalence within Rwandan prisons may genuinely be lower than in the general community, possibly reflecting differences in the strain composition circulating within prison settings. Notably, Rwanda conducts annual active case finding in correctional facilities among both inmates and workers, using TB symptom screening and chest radiography followed by Xpert MTB/RIF testing, which may contribute to early detection before MDR-TB transmission can be established.

We have expanded the discussion to acknowledge these possible explanations.

15. p.13 Line 8 "Importantly, all clusters showed transmission extending beyond Kigali City, with five clusters (1-3, 5, and 6) having only 40-65% of their isolates originating from Kigali province. This highlights the need not only to perform spatial analysis but also to examine transmission dynamics, which may uncover previously undetected transmission patterns." While this observation is interesting, the interpretation that transmission originated from the first notified case should be made cautiously. Without a formal transmission tree reconstruction, the first notified case in a cluster may not represent the true index case especially in tuberculosis transmission.

We agree that caution is warranted when interpreting the directionality of transmission based on the earliest notified case. TB transmission chains are often complex, with long latency periods and potential unsampled cases between notified patients, and we have added this to the Limitations section. However, we wish to clarify that the key observation, that the majority of clusters have 40-65% of their isolates residing outside Kigali, is independent of index case identification and relies solely on the geographic distribution of genetically related cases. This finding robustly supports the conclusion that transmission networks extend beyond Kigali regardless of where transmission originated, and we have revised the text to ensure this distinction is clear.

16. Regarding the discussion of household contact, are there any data showing the proportion of clustered cases with documented household contact? Or if epidemiological investigation data were unavailable, were there cases sharing identical residential addresses? It might make the discussion more evidence-based.

Formal epidemiological investigation data linking cases through household contact were not available in our dataset. While we examined shared residential addresses as a proxy, only a small number of cases within the same cluster shared a residential village, and village-level data does not allow us to distinguish household from community transmission within the same area. These findings highlight the need for future studies incorporating detailed contact tracing data and social network information to better characterise the relative contribution of household versus community transmission in Rwanda.
