# Supplementary material for: Spatial clustering and transmission networks of multidrug-resistant tuberculosis in Rwanda: a national retrospective genomic and spatial epidemiological study
Source: BMJ Open. 2026 Aug 26;16(8):e111823. doi: 10.1136/bmjopen-2025-111823 (PMC13536106; doi:10.1136/bmjopen-2025-111823)
Supplement: Supplementary file 2 [file bmjopen-16-8-s001.docx]

**Supplementary material**

**Table of contents**

[Supplementary tables	2](#_Toc207637766)

[Supplementary figures	6](#_Toc207637767)

**Supplementary tables**

Table S1. A sampling fraction of RR/MDR-TB cases was included in the analysis versus all notified RR/MDR-TB cases. ^*^Only cases with available rpoB sequencing data were included. ^+^WHO has not yet published the official reporting numbers. WHO: World Health Organization. RR-TB: rifampicin-resistant tuberculosis. MDR-TB: multidrug-resistant tuberculosis.

| Year | WHO RR/MDR-TB official notification  n | Included in the analysis^*^  n (%) | Cases with confirmed *rpoB* mutation associated with resistance  n (%) |
| --- | --- | --- | --- |
| 2017 | 79 | 64 (81%) | 48 (75%) |
| 2018 | 97 | 66 (68%) | 35 (53%) |
| 2019 | 101 | 71 (70%) | 36 (51%) |
| 2020 | 40 | 26 (65%) | 23 (88%) |
| 2021 | 35 | 29 (83%) | 24 (83%) |
| 2022 | 61 | 51 (84%) | 27 (53%) |
| 2023 | 92 | 64 (70%) | 25 (39%) |
| 2024 | 68^+^ | 50 (74%) | 35 (70%) |
| Total | **573** | **421 (74%)** | **253 (60%)** |

Table S2. Spatial and spatial-temporal clusters of RR/MDR-TB in Rwanda detected by SaTScan. For the purely spatial analysis, data were aggregated across the entire study period (2017-2024). In contrast, the space-temporal analysis identified a significant cluster during the 2018-2021 period. The "Not applicable" period for the purely spatial analysis indicates that this analysis considered the entire study period without temporal subdivision.

| Cluster number | Time period | Clustering areas, n | Cluster districts | Observed  cases | Expected  cases | Relative  risk | Log likelihood  ratio | *P* value |
| --- | --- | --- | --- | --- | --- | --- | --- | --- |
| Purely spatial | | | | | | | | |
| 1 | Not applicable | 21 | Gahanga, Gatenga, Gatsata, Gikondo, Gisozi, Gitega, Kacyiru, Kagarama, Kicukiro, Kigali, Kigarama, Kimihurura, Kimisagara, Mageregere, Muhima, Niboye, Nyakabanda, Nyamirambo, Nyarugenge, Remera, Rwezamenyo | 101 | 15.82 | 10.06 | 119.95 | < 0.001 |
| Space temporal | | | | | | | | |
| 1 | 2018-2021 | 22 | Gahanga, Gatenga, Gatsata, Gikondo, Gisozi, Gitega, Kacyiru, Kagarama, Kicukiro, Kigali, Kigarama, Kimihurura, **Kimironko**, Kimisagara, Mageregere, Muhima, Niboye, Nyakabanda, Nyamirambo, Nyarugenge, Remera, Rwezamenyo | 62 | 8.49 | 9.39 | 76.21 | < 0.001 |

Table S3. Multivariate binomial regression model of socio-economic and demographic factors influencing the presence of RR-TB hotspots at a sector level in Rwanda, 2017-2024

| Variables | Univariate | | Multivariate | |
| --- | --- | --- | --- | --- |
|  | **Risk ratio (95%CI)** | ***P*** | **Risk ratio (95%CI)** | ***P*** |
| Population density | 1.00 (1.00-1.00) | **< 0.001** | 1.00 (1.00-1.00) | 0.16 |
| Gender ratio (m/f) | 2054.40 (41.82-1.0e5) | **< 0.001** | 4.44 (0.19-104.89) | 0.17 |
| Urban population (%) | 3.2e5 (525.41-2.0e8) | **< 0.001** | 39.78 (0.19-8311.68) | 0.36 |
| RWI | 139.68 (32.29-604.32) | **< 0.001** | 5.81 (0.54-62.10) | 0.15 |
| Investigation rate | 1.00 (1.00-1.00) | 0.12 | - | - |
| Occupancy rate of correctional facilities | 1.00 (0.98-1.02) | 0.96 | - | - |

RWI: Relative Wealth Index. P: p-value

Table S4. Demographic and clinical characteristics of 201 patients included in the high-resolution molecular epidemiology study of multidrug-resistant tuberculosis transmission in Rwanda, stratified by genomic cluster status (≤5 SNP distance), 2017–2024.

| Category | Cluster and lineage | | | | | | Ungrouped  n=92 |
| --- | --- | --- | --- | --- | --- | --- | --- |
|  | **1, n=19** | **2, n=31** | **3, n=10** | **4, n=11** | **5, n=15** | **6, n=23** |  |
|  | **4.6.1.2** | **4.6.1.2** | **4.6.1.2** | **4.6.1.2** | **4.6.1.2** | **4.7** |  |
| Gender  Male  Female  Uknown | 13 (68%)  6 (32%)  0 | 21 (68%)  10 (32%)  0 | 7 (70%)  2 (20%)  1 (10%) | 9 (82%)  2 (18%)  0 | 9 (60%)  6 (40%)  0 | 16 (70%)  7 (30%)  0 | 70 (76%)  22 (24%)  0 |
| Age  Median [IQR] | 35 [31-41] | 35 [28-42] | 38 [33-44] | 33 [25-38] | 38 [29-53] | 35 [29-45] | 38 [27-46] |
| HIV status  Positive  Negative  Unknown | 4 (21%)  14 (74%)  1 (5%) | 10 (32%)  20 (65%)  1 (3%) | 5 (50%)  4 (40%)  1 (10%) | 4 (36%)  6 (55%)  1 (9%) | 3 (20%)  12 (80%)  0 | 8 (35%)  15 (65%)  0 | 28 (30%)  64 (70%)  0 |
| TB history  New case  Retreatment  Unknown | 13 (68%)  6 (32%)  0 | 23 (74%)  7 (23%)  1 (3%) | 8 (80%)  1 (10%)  1 (10%) | 8 (73%)  3 (27%)  0 | 12 (80%)  3 (20%)  0 | 21 (91%)  2 (9%)  0 | 66 (72%)  23 (25%)  3 (3%) |
| Province  Kigali City  East  North  South  West | 10 (53%)  4 (21%)  1 (5%)  3 (16%)  1 (5%) | 18 (58%)  6 (19%)  0  5 (16%)  2 (6%) | 6 (60%)  2 (20%)  0  2 (20%)  0 | 9 (82%)  0  0  0  2 (18%) | 6 (40%)  3 (20%)  2 (13%)  3 (20%)  1 (7%) | 15 (65%)  0  0  7 (30%)  1 (5%) | 35 (38%)  17 (19%)  7 (8%)  19 (21%)  13 (14%) |
| Isoniazid  Susceptible  Resistant  Unknown | 0  19 (100%)  0 | 0  30 (97%)  1 (3%) | 0  10 (100%)  0 | 0  11 (100%)  0 | 0  15 (100%)  0 | 0  23 (100%)  0 | 16 (17%)  76 (83%)  0 |

**Supplementary figures**

Figure S1. A) Case notification rate of confirmed rifampicin-resistant tuberculosis (RR-TB) patients diagnosed between 2017 and 2024 per 10,000 inhabitants in the 416 administrative sectors of Rwanda. B) Population density (inhabitants per km2) per sector. Source data: 5^th^ Rwanda Population and Housing Census 2022.

Figure S2. Map showing the sectors included in spatial clusters of RR-TB patients in Rwanda (2017-2024). Red sectors indicate areas included in both the purely spatial cluster (entire study period) and the space-temporal cluster (2018-2021). The orange sector (Kimironko) was included only in the space-temporal cluster but not in the purely spatial cluster.

Figure S3. Spatial K-function difference analysis of six MDR-TB genotypic clusters compared with ungrouped strains in Rwanda, 2017-2024. Values above the 95% confidence envelope represented in grey indicate significant spatial clustering.
